# On the hierarchical inheritance of aftereffects in the visual system

**DOI:** 10.3389/fpsyg.2013.00472

**Published:** 2013-08-01

**Authors:** J. Edwin Dickinson, David R. Badcock

**Affiliations:** Human Vision Laboratory, School of Psychology, University of Western AustraliaPerth, WA, Australia

**Keywords:** tilt aftereffect, shape aftereffect, face aftereffect, tilt aftereffect field, adaptation

## Abstract

The emotion perceived in a face can be influenced by prior exposure to a face expressing a different emotion. Here we show that displacement along a particular emotional axis, that encoding happiness and sadness, can be effected solely by a systematic change in the angle, at the center of the mouth, between the left and right halves of the mouth. We then demonstrate that adaptation to a face with the mouth distorted to change this angle, such that the face expresses an emotion on this axis, causes a face with a neutral expression to be perceived as having the opposite expression. By abstracting the mouths from the faces and examining the magnitude of the angle aftereffects in the mouths alone and in an unfamiliar orientation, we show that the magnitudes of the angle aftereffects are sufficient to account for the changes in perceived emotion in the faces. Further, by applying the distortion to the mouths asymmetrically so that the distortion is manifested by a change in orientation of the mouth stimulus rather than a change in angle, we show that the magnitude of the aftereffect can be predicted by the local tilt aftereffect. We argue, therefore, that the aftereffects of emotion are due to misperception of morphology of the face and that the misperception is due to the local change in perceived orientation due to the systematic application of the tilt aftereffect in a tilt aftereffect field. All adaptation experiments were performed using stimuli that were either high-pass or low-pass filtered for spatial frequency. Results showed that the spatial frequency specificity of the aftereffects was the same for the face, angled mouth, and oriented mouth stimuli, lending further support to the hypothesis that the aftereffects are instantiated in processes early in the visual cortex and that the aftereffects assumed to be higher level are, in fact, inherited.

## Introduction

Concerning the study of the functionality of mechanisms of the brain a frequently cited aphorism is that aftereffects represent the psychologist's microelectrode (Frisby, 1979). The justification for this comparison is the similarity between the neuro-physiologically derived functions describing the response of single neurons and the perceptual deficits introduced by adaptation. An example is the function describing the response of neurons of the primary visual cortex to a line as its orientation is varied, and orientation specific deficits in sensitivity to gratings revealed in psychophysical tasks after adaptation to gratings of a particular orientation. The aftereffect, a perceptual deficit in this instance, mirrors the decline in neuronal sensitivity [comprehensive recent reviews of potential neural mechanisms of adaptation are provided by Kohn (2007) and Clifford et al. (2007)]. The stimulus selectivity of the visual system revealed by this particular aftereffect is known as an orientation channel (Graham, 1989) and sensitivity to the whole range of orientations is afforded by a set of channels with differing preferred orientations. Such is the utility of adaptation in the demonstration of tuning of the visual system to particular stimuli that it has become the method of choice for inferring the algorithmic units of vision in the absence of neuro-physiological data. For example, adaptation to a sinusoidal grating of a particular orientation results in a notch in the graph describing contrast sensitivity to the same grating as a function of orientation (Gilinsky, 1968; Blakemore and Campbell, 1969) but not in that of a grating with a substantially different frequency (Blakemore and Campbell, 1969). This leads to the assumption that, on a local level, the visual system is tuned for gratings of a particular orientation and spatial frequency. Significantly, however, the effect of adaptation to a grating is observed even if the point of fixation is allowed to move freely around the adapting pattern, resulting in a homogeneous adaptation of a region of the visual field to the oriented grating [Arend Jr and Skavenski (1979) showed that observers preferentially fixate certain phases of gratings of particular spatial frequencies, but also that the fixation on the preferred phase was on average less than twice that of any other phase. Given the logarithmic nature of the time-course of adaptation, this inhomogeneity in the duration of adaptation would be only weakly reflected in the state of adaptation. Different observers exhibited different preferred phases]. This observation suggests that the sensitivity that is lost is sensitivity to lines or boundaries of a particular orientation and spatial scale. The receptive fields of neurons of the primary visual cortex are well described by oriented spatial weighting functions with regions of excitatory and inhibitory response to light (Hubel and Wiesel, 1977; Kulikowski et al., 1981; Field and Tolhurst, 1986). The notch in the contrast sensitivity function post adaptation can be understood as due to a reduction in sensitivity of those neurons whose receptive fields approximated the luminance profile of the adapting grating, for some period of the adapting interval, and were therefore stimulated by the grating.

At this point it is instructive to consider how the local orientation of extended features of a stimulus might be represented. The responses from the receptive fields of the simple cells in the primary visual cortex are not unique to particular stimuli and, therefore, it has long been recognized that they cannot represent specific feature detectors (Marr, 1976). For example, certain cells of the primary visual cortex have receptive fields with excitatory and inhibitory areas adjacent along a boundary and, thus, respond to a change in luminance at an edge. An inappropriately oriented but high contrast edge could, therefore, elicit the same response as a more appropriately aligned edge of lower contrast. The visual system resolves this ambiguity by sampling small regions of the visual field over the whole range of orientations. Because these samples are all subject to the same local contrast environment, the cell with the orientation that most closely matches the orientation of the edge would give the largest response. Judgment of orientation is, however, more precise than would be inferred from the orientation tuning of a single cell (Westheimer et al., 1976; Jastrow, 1892; Westheimer, 1990). It has, therefore, been proposed that the perceived local orientation is determined within a population of orientation selective cells (Westheimer, 1990), perhaps by the centroid of the response of a population of orientation selective cells that span the whole orientation spectrum. This form of explanation was first used to explain a repulsion in perceived auditory frequency from the frequency of an adapting tone by Georg von Bekesy (Bekesy, 1929). The population of cells that samples the orientations in the same local region is clustered within a volume of cortex known as a hypercolumn. A hypercolumn is subdivided into columns perpendicular to the cortical sheet with each column containing neurons with receptive fields of a particular orientation selectivity (Hubel et al., 1978). The preferred orientation changes systematically across the hypercolumn and, because the orientation tuning of a neuron is broad in comparison with the incremental change in orientation selectivity across columns, the response to an oriented feature extends across a number of adjacent columns. One can envisage this distribution of activation as a histogram of activity on the cortical sheet, but how might this distribution represent a particular orientation? A model proposed by Gilbert and Wiesel (1990) represented neuronal responses of the orientation selective cells as vectors in the Cartesian plane and the perceived orientation as the vector sum of these vectors. A horizontal line is, however, as different in orientation from a vertical line as is possible. Similarly a line at −45° to the vertical is as different as possible in orientation from a line at 45° to the vertical. If, therefore, we represent orientation in a Cartesian reference frame with vertical and horizontal on the positive and negative y axis, respectively and 45° and −45° on the positive and negative x axis, respectively then orientation is uniquely represented as a vector in this double angle space (Clifford, 2002). The preferred orientations of the orientation columns can be represented as vectors in this space and a line of a particular orientation can then be represented by the vector sum of the response of all orientation columns of a hypercolumn. This model assumes, of course, that the components of each vector can be encoded in some way to allow the vector summation. The model does not speculate on how this might be achieved neuronally but a natural consequence of this representation is that adaptation to a particular orientation, resulting in a reduction in sensitivity to that orientation, causes the resultant vector representing a test line to be repelled from the orientation of the adapting line. From a mechanistic point of view the aftereffect is due to a displacement in the centroid of the response of a bank of orientation selective channels due to modification of the relative sensitivities of the channels by prior adaptation to a specific orientation. A reduction in the sensitivity of the channels stimulated by the adaptor leads to a repulsion of the centroid from the adapting orientation. Such repulsion is indeed observed and is known as the tilt aftereffect (Gibson, 1937). Clifford et al. (2001) showed that this model can account for the tilt aftereffect observed for hard edged circular windowed gratings and Dickinson et al. (2012a) subsequently showed that it can predict the observed magnitude of the tilt aftereffect as a function of the orientation difference between the adapting and test orientations of groups of Gabor patches.

In the previous paragraph we have seen that if a particular mechanism for representation of orientation is accepted, then an explanation for the tilt aftereffect naturally follows. The aftereffect is a misrepresentation of orientation, a purely geometrical property, and the explanation we have provided for it is feed forward. This explanation links the tilt aftereffect to variations in neuronal sensitivity within a particular volume, a hypercolumn, of the primary visual cortex. A hypercolumn deals with a small region of the visual field with neighboring hypercolumns dealing with adjacent regions. Since the primary visual cortex is retinotopically arranged, that is mapped to the retina in a manner that preserves spatial order across the visual field, the tilt aftereffect experienced in any particular region of the visual field will be determined by the difference in orientation between adaptor and test in the corresponding region of the retina (Knapen et al., 2010). Recognition that orientation can be misperceived locally due to adaptation, however, begs the question of how extended objects might be misperceived. In an elegant adaptation experiment Blakemore and Over (1974) showed that if a curved adapting grating, concave to the right, was scanned repeatedly along the horizontal midline then a subsequently viewed straight vertical line was perceived as concave to the left; but if the adapting grating was scanned along the vertical midline then the line appeared undistorted. In the first instance the region of cortex in spatial correspondence with the top half of the test stimulus becomes adapted to an orientation anticlockwise of vertical and the bottom half clockwise of vertical. In the second the top and bottom halves are similarly adapted to orientations both clockwise and anticlockwise of vertical. Blakemore and Over concluded that this apparent adaptation to curvature was consistent with a systematic application of the tilt aftereffect, and indeed it is consistent with the mechanism proposed above to explain the tilt aftereffect. For the first adapting method the resultant vector representing the orientation of the vertical, linear test grating would be anti-clockwise of vertical for the top half of the stimulus and clockwise of vertical for the bottom. For the second adapting method the resultant vector would be vertical for all regions of the test stimulus because the adaptation is symmetrical about the vertical. The results of this experiment are, therefore consistent with a locally constrained adaptation combined with the accumulation of adaptation across eye movements.

Dickinson et al. (2010) proposed a general mechanism to account for shape aftereffects based on the tilt aftereffect. They postulated that shape aftereffects could be predicted by a systematic application of the tilt aftereffect across the stimulus, concomitant with a misrepresentation of the locus of extended features to preserve continuity of those features. This mechanism was shown to predict the selective misperception of a coincident circle and Cartesian grid after adaptation to a radial frequency (RF) pattern, a pattern deformed from circular by a sinusoidal modulation of radius, or a Cartesian grid deformed in the same manner. Dickinson et al. (2012b) went on to show that the adaptation was retinotopic and rapidly acquired as would be predicted by a retinotopically constrained (Afraz and Cavanagh, 2008; Knapen et al., 2010) and rapidly induced (Sekuler and Littlejohn, 1974) tilt aftereffect. The representation of the tilt aftereffect extended over space was referred to as a tilt aftereffect field. The tilt aftereffect field is a scalar field which represents the tilt aftereffect at any point in the visual field, determined locally by the orientation difference between the adapting stimulus and the test stimulus. It is easy to imagine how complexities in this representation might arise. The null adaptation result of Blakemore and Over, however, is readily accommodated by allowing the adaptation to be accumulated over time resulting in a null tilt aftereffect field when the orientation channels with preferred orientations clockwise and anticlockwise of the test orientation are symmetrically adapted. Another problem that was identified in Dickinson et al. (2010) was that when lines of different orientations intersect they are likely to be subject to different tilt aftereffects due to the same adaptation history. This problem was circumvented by treating the lines close to horizontal and vertical as being subject to separate tilt aftereffect fields [cells of V1 do not respond to orientations perpendicular to their preferred orientation (Ringach et al., 2002)]. Thus, the simplicity of the tilt aftereffect field representation of shape aftereffects is somewhat compromised for complex stimuli but the general principal, that shape aftereffects are due to the systematic application of the tilt aftereffect is in no way invalidated. Dickinson et al. (2010) therefore proposed that the tilt aftereffect field explanation for shape aftereffects would generalize across all extended visual stimuli and should be entertained as a possible explanation for all such aftereffects. Because the tilt aftereffect field is simply a representation of the tilt aftereffect over an extended area, the shape aftereffects due to application of this field require no recourse to the heuristic influences intrinsic to the visual system that are used to make sense of scenes. The aftereffects would be, therefore, purely a consequence of morphological differences between the adapting and test stimuli. Aftereffects due to adaptation to semantic information, however, can reasonably be expected to act at the level of internal representations of the world.

The face might be considered one of the most evocative visual stimuli, suffused with ecologically relevant information. A large proportion of such information is morphologically signaled, and arbitrary geometrical transformations of face shape are seen to act on identity (Blanz et al., 2000). The morphology of a specific face is different from that of a face with a morphology representing the mean of a population of faces. Faces, then, can be specified by the geometrical transformation required to transform the mean face to the specific face. If the opposite transformation is applied to the mean face an anti-face with a distinct identity results. When adapted to the anti-face, however, an observer is more inclined to identify the mean face as the original specific face, than when un-adapted (Leopold et al., 2001; Wilson et al., 2002; Rhodes and Jeffery, 2006). Within a particular identity, though, particular changes in the morphology of the face signify changes in emotional state and if these emotional states are to be useful within a population of individuals then the processing of such differences must generalize across identities. In a recent study Skinner and Benton (2010) created anti-expressions in faces using the process of applying a geometrical transformation opposite to that required to produce the expression from a face with a neutral expression. The expressions manipulated were happiness, sadness, fear, anger, disgust, and surprise. After adaptation to a face with an anti-expression, observers were required to report which of the six expressions was perceived in a neutral face. In the majority of trials, the observers reported the expression opposite to the anti-expression. For example, they reported fear in a neutral face after exposure to a face with an anti-fear expression. Both identity and expression aftereffects, therefore, are recognized to be consistent with geometrical transformations opposite in sign to the transformations applied to the test patterns to create the adaptors. The effects of adaptation are assumed by the authors of these studies to act at the levels of visual processing associated with the analysis of faces, and claims are made regarding the nature of such analyses on the basis of the selectivity of adaptation effects. It is, however, possible that the adaptation takes place earlier in the visual processing hierarchy and Dickinson et al. (2010) showed that adaptation to an arbitrarily transformed face produced the percept of the opposite transformation in an untransformed face. Naturally this result would also be expected to apply to transformations of faces that conferred meaning. Moreover, the relationship between the magnitude of the aftereffect and the size of the transformation of the adaptor revealed by Dickinson et al. (2010) was the same as that for the same transformation introduced into a circle, which was shown to be consistent with the application of a tilt aftereffect field. Although this result shows that the aftereffect might be consistent with the application of a tilt aftereffect field, a face is a much more complex stimulus than a circle and so it is more difficult to demonstrate this explicitly. It is, though, possible to test some further predictions of this interpretation.

**Figure 1:**
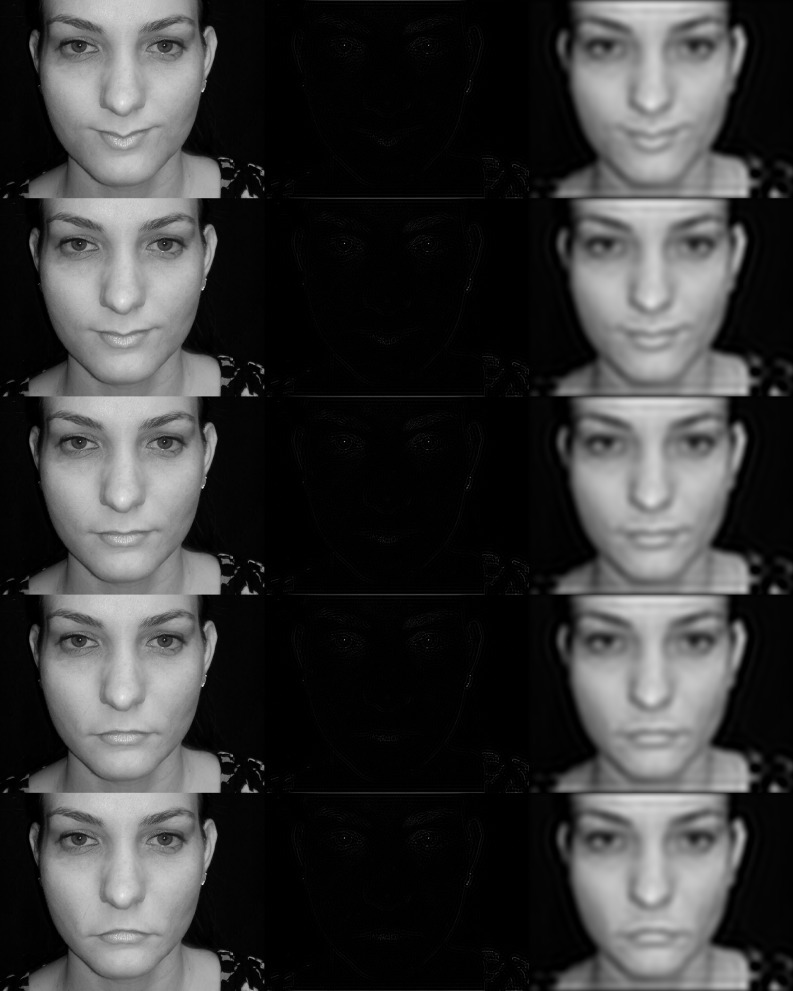
**Example face stimuli**. The left hand column shows, from top to bottom, faces with angles of +15, +8, 0, −8, and −15 degrees introduced to the mouth as described in the material and methods section. The third face from the top in the left hand column is cropped from the original photograph of VB taken by Matt Tang of the Human Vision Laboratory of the University of Western Australia. The middle and right hand columns are high-pass and low-pass filtered versions of the same faces, respectively. From top to bottom the faces change from appearing happy to sad. The faces with +15 and −15 degree deformations in angle introduced to the mouth were used as adapting stimuli. Those with +8 and −8 deformation in angle were the extreme ends of the spectrum of test stimuli used in the method to constant stimuli to determine the point of subjective equality, the face which observers would report as happy or sad with equal probability. The high pass filter stimuli in the middle column retain the same high spatial frequency energy as in the original images. Removing the low frequencies reduces the overall mean luminance in the face and therefore these images are best viewed by zooming in on the sub-images, although they are depicted as used in the experiment.

**Figure 2:**
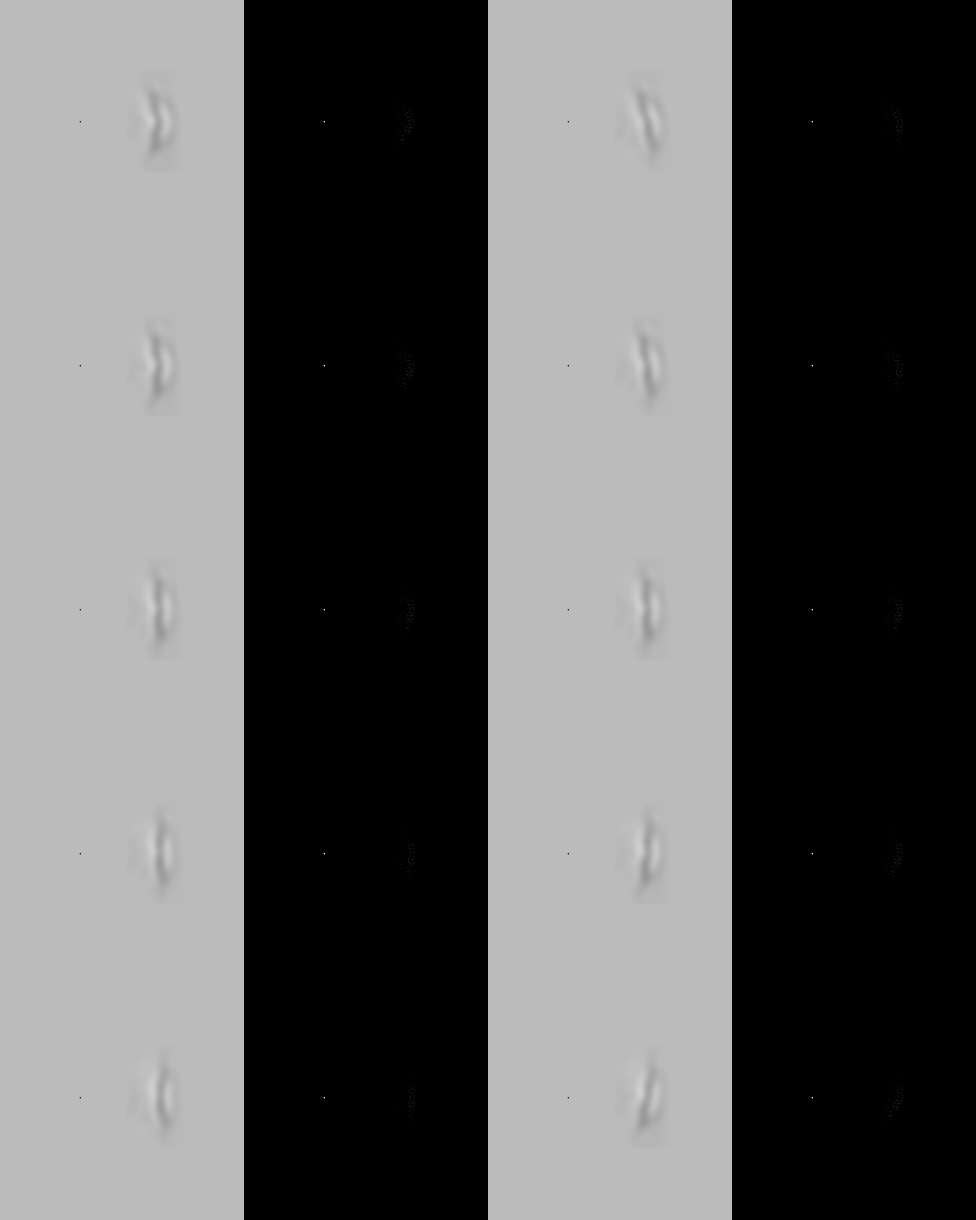
**Example angled and oriented mouth stimuli**. The two columns on the left half of the figure show mouths abstracted from the faces and turned anticlockwise through 90°. The deformations in angle in these examples are +15, +8, 0, −8, and −15 degrees above the horizontal midline of the mouth. The columns in the right hand half of the figure show mouths with the opposite displacement applied to the right and left halves of the mouth to create mouths that appear oriented. The pairs of angles introduced to the right/left halves of the mouth, in these examples are +15/−15, +8/−8, 0/0, −8/+8, and −15/+15 degrees above the horizontal midline of the mouth (before the mouth was rotated). For these stimuli only the low-pass and high-pass spatial frequency versions of the stimuli are shown.

If the adaptation is manifested at the higher levels of visual processing associated with analysis of faces, then the aftereffects should be dependent on the semantic information content of the face. If it is simply due to a tilt aftereffect field, however, then it should depend on the local feature properties of the stimulus. A system incorporating some redundancy might display both local morphological and semantic adaptation. Xu et al. (2008) showed that adaptation to a curve can influence the perception of high level facial expressions and Benton (2009) used the folded face illusion to show that introducing a vertical shear in a neutral face could cause the face to appear happy or sad. Dickinson et al. (2012b) showed that adaptation to a local orientation field that might be expected to introduce this shear, as a result of the misperception of the orientation of the features orientated close to the horizontal, can cause a subsequently viewed un-manipulated face to have the opposite demeanor. That is, the aftereffects of adaptation to the orientation fields cause the orientations of the face to undergo the same transformations as they do in the folded faces. As we shall show, simply introducing shear, effectively an angle, solely into the mouth of an adaptor face can produce the same emotion aftereffects. Restricting the manipulation to the mouth allows us to then abstract the mouth from the face to test whether adaptation to the angle introduced into the mouth can produce a perceived angular change in a linear mouth sufficient to account for the perceived change in demeanor of the face. For this manipulation the mouth is presented rotated through a right angle to require the judgments to be made at a mouth orientation that is experienced less frequently than in the horizontal. In a further manipulation of the abstracted mouth, we apply the opposite transformations to the two sides of the mouth (that is the transformation of the two sides of the mouth result in clockwise or anti-clockwise rotations of both sides of the mouth about the center of the mouth) to create adapting and test stimuli that are essentially linear but effectively rotated, to determine if adaptation to a rotated, or tilted, mouth can produce a perceived rotation in a mouth sufficient to account for the previous two manipulations (see Figures 1, 2). The adapting and test mouths are again presented rotated anti-clockwise through 90 degrees so that the orientations of the test mouths span the vertical. By requiring the observers to report whether manipulated faces appear happy or sad in an unadapted condition we demonstrate that the demeanor of the faces can be reliably reported for both the high- and low-pass filtered stimuli which then allows us to examine cross adaptation between these two stimulus types. If adaptation due to the semantic content of the faces occurs then adaptation should be strongly evident for the face stimuli but somewhat reduced for the angled mouth and oriented mouth stimuli because the semantic information is present in the face stimuli but not in the angled mouth and oriented mouth stimuli. If, though, the adaptation occurs solely at the local level then adaptation should be similar across the three stimulus types (face, angled mouth, and oriented mouth). In addition to these spatial manipulations of mouth shape the face and mouth stimuli are high-pass and low-pass filtered for spatial frequency. It has been shown that the tilt aftereffect is selective for spatial frequency (Ware and Mitchell, 1974) and that inter-ocular transfer of the aftereffect is correlated with stereo-acuity (Mitchell and Ware, 1974). The second of these studies demonstrated an absence of transfer in stereo-blind subjects. Collectively these two investigations suggest that the locus of the mechanism supporting the tilt aftereffect is earlier than the convergence of the information from the two eyes onto binocularly driven neurons. If, then, the face and angle aftereffects observed in the face and angled mouth stimuli are simply the effects of a tilt aftereffect field we can reasonably expect that the aftereffects would be larger for adapting and test stimuli matched in spatial frequency than for stimuli with differing spatial frequency, and also that the difference in the sizes of the effects would be the same for the oriented mouth, angled mouth and face stimuli. Differentially selective aftereffects for the different stimulus types might, however, be indicative of the presence of a higher level (semantic) aftereffect.

## Materials and methods

Four experienced psychophysical observers ED, MT, RG, and TM, all with normal or corrected to normal visual acuity, participated in the experiment. The experiment complied with the requirements of the University of Western Australia research ethics committee and was therefore conducted in accordance with the Declaration of Helsinki. ED is an author and informed consent for participation in the experiment was obtained from MT, RG and TM.

Stimuli were presented on a Sony G520 monitor from the frame buffer of a Cambridge Research Systems Visage visual stimulus generator. The monitor was luminance calibrated using a CRS Optical and associated software at a refresh rate of 100 Hz. Adaptor and test stimuli were each presented for durations of 160 ms with a 500 ms inter-stimulus interval interposed. Screen luminance during the inter-stimulus interval, before the adapting interval and after the test interval was 9 cd/m^2^. The screen was viewed from a distance of 135 cm, at which distance each screen pixel subtended 1 min of visual angle.

A member of the Human Vision Laboratory, VB, posed for a photograph whilst displaying a neutral expression, neither happy nor sad. The image of the face was composed of 1024 × 768 pixels with a mouth width of approximately 270 pixels, or 270 min of visual angle when presented in the experiment. In order to create the happy and sad faces from this image the horizontal midline of the mouth was displaced vertically upwards or downwards, respectively, by the tangent of a specified angle multiplied by the distance from the center of the mouth out to a distance of 157′. The vertical displacement beyond this distance was returned linearly to zero over the next 40′. Pixels above and below the midline were moved by the same amount scaled by a Gaussian profile in the vertical with a standard deviation of 50′ to allow a smooth transition into the undistorted region of the face (see Figure 1). The faces used for the adaptor stimuli had displacements equating to a rotation of the horizontal midline of the mouth about the center of the mouth by angles of 15 degrees above (positive) and below (negative) the horizontal applied to the neutral face to create faces that appeared very happy and very sad, respectively. This manipulation will be referred to as a displacement in angle with the convention for faces that a positive displacement in angle results in a smiling face. Nine faces were used to create test stimuli with displacements equating to angles of −8, −6, −4, −2, 0, +2, +4, +6, and +8 degrees. High- and low-pass filters were applied to all of these faces. The high-pass filters removed all spatial frequencies of less than 3.6 c/° and the low-pass filters removed frequencies of greater than 1.8 c/°. A band of spatial frequencies with a range of an octave was, therefore, completely removed from the stimuli and separates the frequency content of the high- and low-pass stimuli. In order to verify that the test faces were perceived to express happiness and sadness observers were asked, in an unadapted condition, to report whether they perceived the test faces as happy or sad. The test faces were reliably reported as happy if they had a positive displacement of the mouth and sad if they had a negative displacement (essentially 100% reliable at the two extremes of the range of stimuli used for the test faces).

To create the angled mouth stimuli a two dimensional Gaussian luminance contrast window was applied to the face stimuli centered on the middle of the mouth in order to smoothly match the luminance of the abstracted mouth to a flat background luminance. The background luminance of the low-pass stimuli was 63 cd/m^2^ and the high-pass stimuli 0 cd/m^2^. The standard deviation of the horizontal axis, of the window was 50′ and the vertical axis 25′. Following the application of this window a rectangular area of 320 × 190 pixels, centered on the midpoint of the mouth was abstracted and pasted into flat images with the appropriate background luminance. The mouth was rotated anti-clockwise through 90 degrees and presented four degrees of visual angle to the right of a fixation point at the center of the screen (see Figure 2). No fixation mark was used for the face stimuli but observers were instructed to fixate the bridge of the nose which was approximately four degrees of visual angle above the mouth. Across the stimulus types, therefore, the mouths were at an eccentricity of four degrees. The same convention of displacement in angle applies to the angled mouth stimuli but, as the mouths are rotated anti-clockwise through a right angle, positive displacements in angle result in mouths that are concave to the left.

The oriented mouth stimuli were created from the mouth stimuli by matching the top halves of the mouth stimuli (actually the right half of the mouth) with positive displacements to the bottom halves of the stimuli with negative displacements and vice versa. An oriented mouth stimulus with an anti-clockwise tilt of 8 degrees, for example, would have the top half of a mouth with a +8 degrees displacement and the bottom half of a mouth with −8 degrees displacement. The convention for the oriented mouth stimuli is that a positive displacement in angle is an anti-clockwise rotation. These stimuli were again presented 4 degrees of visual angle to the right of a centrally located fixation point. Example face stimuli are presented in Figure 1 and example angled and oriented mouth stimuli in Figure 2.

Each of the three stimulus types (face, angled mouth, oriented mouth) were filtered to give low-pass (LP) and high-pass (HP) filtered versions of the stimuli. For each condition the method of constant stimuli (MOCS) was used to determine the point of subjective equality (PSE); a neutral expression for the faces (neither happy nor sad), a straight mouth for the angled mouth stimuli and a vertical mouth for the oriented mouth stimuli for the LP and HP versions of the three stimulus types. For each of the test stimuli (LP and HP filtered face, angled mouth and oriented mouth stimuli) the PSE was determined in the absence of an adaptor and under four conditions of adaptation to the same stimulus type, the four conditions being LP and HP stimuli with positive (+15 degrees) and negative (−15 degrees) displacements in angle, as previously defined. To summarize, there are six test stimuli, comprising LP and HP filtered versions of the face, angled mouth and oriented mouth stimuli. Adaptation was restricted to similar stimulus types (faces with faces for example) and the points of subjective equality determined for adapting stimuli with positive and negative deformation and with similar and dissimilar filter conditions. For each condition (adaptor—test pair), three blocks of 180 trials were performed. For all conditions the test stimuli used in the MOCS were divided equally across stimuli with −8, −6, −4, −2, 0, +2, +4, +6, and +8 degrees of displacements in angle. These stimuli ranged from sad to happy for the faces, concave to the right to concave to the left for the mouth stimuli and clockwise of the vertical to anti-clockwise of the vertical for the orientated mouth stimuli. On each trial the observer was required to report whether the face appeared happy or sad, whether the mouth stimulus appeared concave to the left or right or whether the oriented mouth stimulus appeared oriented anti-clockwise or clockwise of vertical using the left or right mouse button respectively. The test stimuli comprised HP or LP filtered stimuli of the three stimulus types and for each of these a no adaptor condition and HP and LP adaptor conditions (each with conditions with +15 and −15 displacements in angle) were performed using the same test stimulus type. The probabilities of reporting that the test stimuli were happy, concave to the left or anti-clockwise of vertical were calculated, as appropriate, for each level of displacement. A cumulative normal distribution was fitted to the probabilities with the mean yielding the displacement in angle for the stimuli required to give the PSE, that is; a neutral expression for the faces, a straight mouth for the angled mouth stimulus and a vertical oriented mouth stimulus, respectively. Afterwards aftereffect magnitudes were derived by taking difference between the PSEs for positive (+15) and negative (−15) displacements in angle for each adaptor condition (for example the difference between the points of subjective equality for a high-pass filtered test face after adaptation to low-pass filtered faces with positive and negative displacements in angle applied to the mouths). These aftereffect magnitudes were used for statistical testing.

## Results

**Figure 3:**
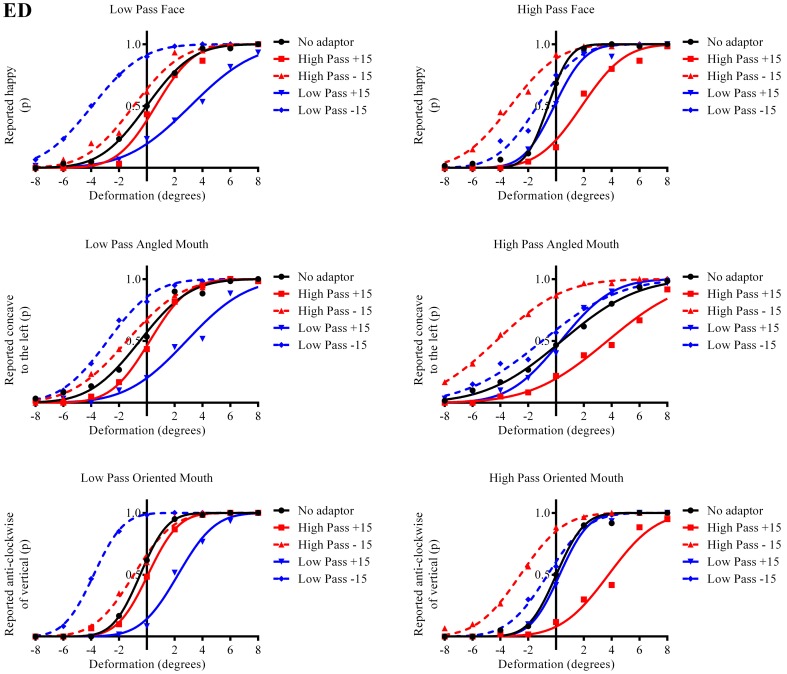
**Psychometric functions for Observer ED**. The three rows of graphs present data for the three stimulus types; faces, angled mouths and oriented mouths from top to bottom. Adaptation was examined within but not across these stimulus types but the same sizes of deformation in angle were used in the mouths of the adapting stimuli in each case (equating to an introduction of either +15 or −15 degrees of rotation of the two halves of the mouth about the center of the mouth and with respect to the midline of the mouth). The left column of graphs displays the data for the low-pass (LP) spatial frequency test stimuli and the right column the high-pass (HP) test stimuli. Each graph, therefore, represents data for a single test stimulus (top left is the data for a LP test face, for example, and this information is used as the label for each graph). For each test stimulus four adapting conditions were examined; these being positively (+15 degrees of deformation in angle) and negatively (−15) deformed LP and HP versions of the same stimulus type. The red lines represent the fitted psychometric functions for the HP spatial frequency adaptors and blue lines LP. Solid lines represent the psychometric functions for positively deformed adaptors (+15) and dashed lines negatively deformed (−15). The solid black lines are the functions fitted to the data from the un-adapted conditions. As an example of the convention for the representation of data, the dashed blue line in the top left graph represents the probability of responding that the LP test face appears happy, after adaption to a LP adapting face with a deformation in angle of −15 degrees applied to the mouth, as a function of the deformation in angle applied to the test face. In this example it is clear that adapting to the negatively deformed adapting face (which appears sad) results in the observer reporting that the test face appears happy more frequently for all amplitudes of test face deformation. The point of subjective equality (PSE), the point at which the observer reports happy and sad with equal probability is displaced toward negative values for deformation in angle of the test face. That is, a test face must be deformed toward sad in order to appear neutral after adaptation to a sad face. In all cases it is evident that after adaptation the stimuli perceived as untransformed are in fact transformed in the direction of the adaptor. This transformation is required to null the aftereffects of the adaptation. For this observer the aftereffects of adaptation are much larger for the adaptor and test stimuli with similar spatial frequency content than for those that are dissimilar.

**Figure 4:**
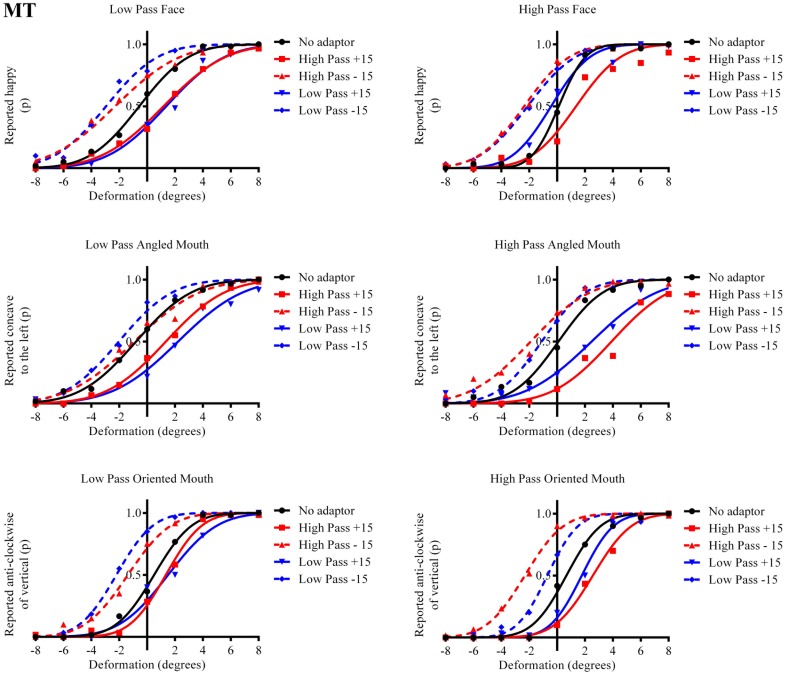
**Psychometric functions for Observer MT**. The format of these graphs is the same as those for Observer ED in Figure 3. The one substantial difference from the data for Observer ED is that there is a smaller difference in the magnitude of the aftereffect between the conditions where the adaptor and test stimuli had the same and different spatial frequency content. The aftereffects of adaptation, though, are still largest for the stimuli with similar spatial frequency content.

**Figure 5:**
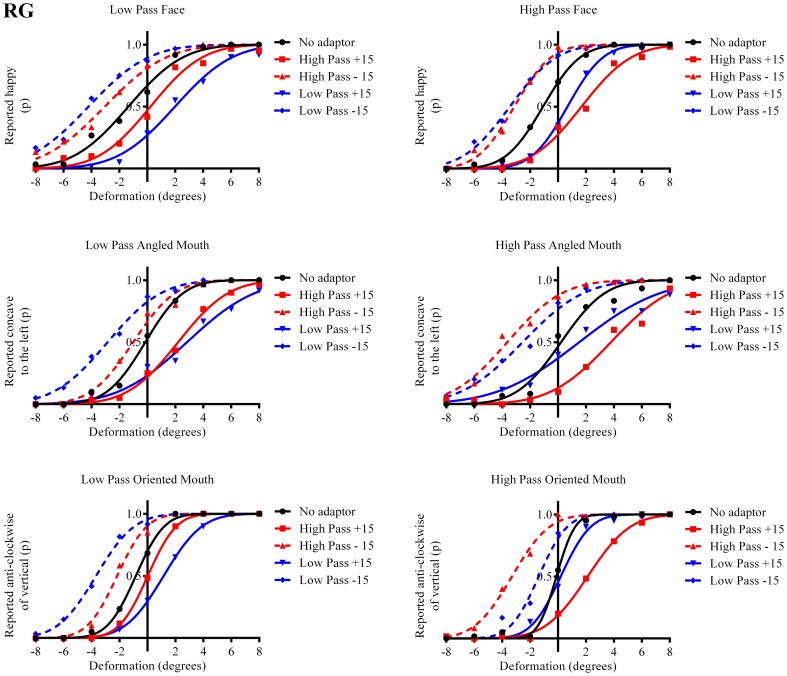
**Psychometric functions for Observer RG**.

**Figure 6:**
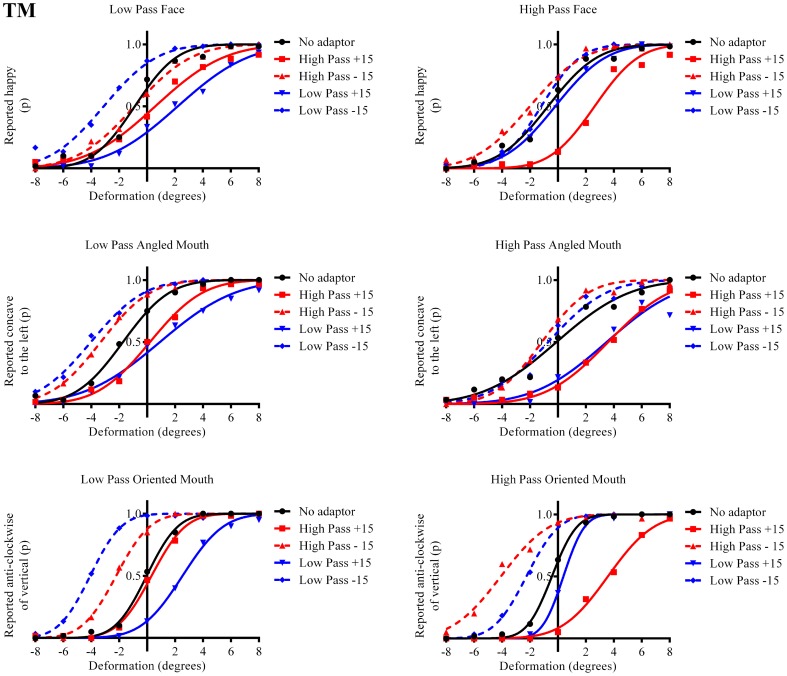
**Psychometric functions for Observer TM**.

The results in the form of psychometric functions are presented in Figures 3–5 and 6 for observers ED, MT, RG, and TM, respectively.

**Figure 7:**
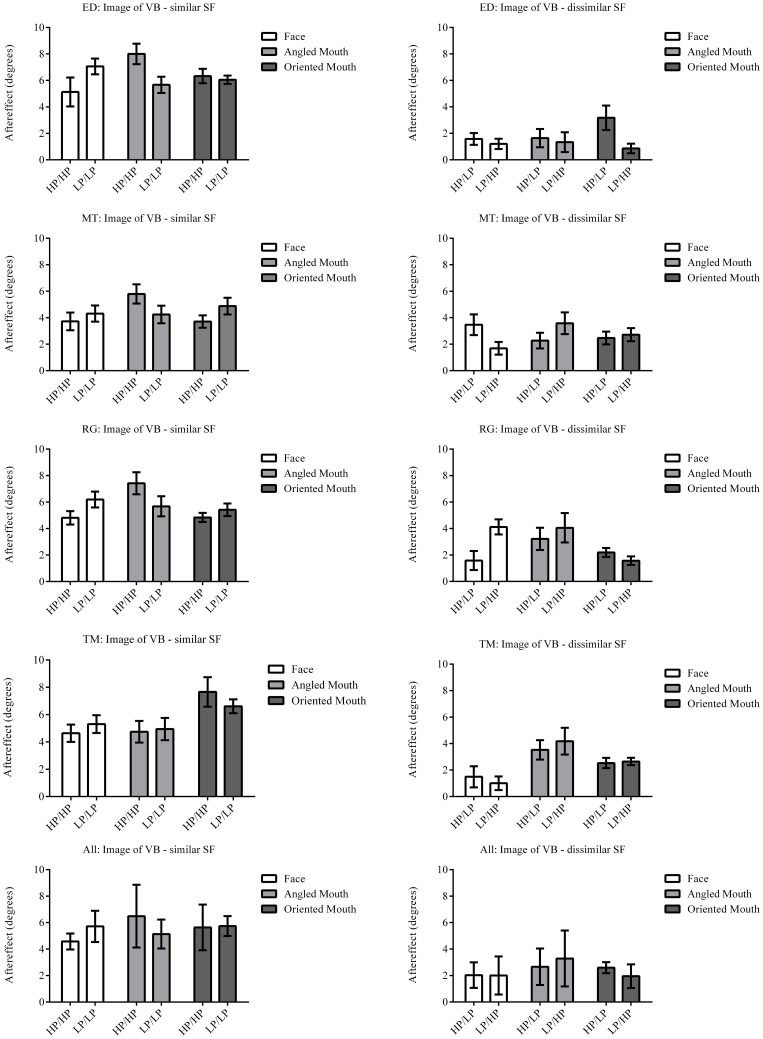
**Aftereffect magnitudes for Observer ED**. The aftereffect magnitudes displayed are the differences between the points of subjective equality for the same adaptor (in terms of stimulus type and spatial frequency) with positive and negative deformations in angle. For example the difference between the points of subjective equality for the low-pass filtered face after adaptation to high-pass filtered faces with deformations in angle of +15 and −15 degrees is represented by the column filled in white (labeled “Face” in the legend) and annotated HP/LP in the bottom graph. The magnitude of the aftereffect is similar across face, angled mouth and oriented linear mouth stimuli for conditions that have the same spatial frequency content in adaptor and test (left column of graphs), and also for conditions that have dissimilar spatial frequency content in adaptor and test (right column of graphs). Conditions with similar spatial frequency content in adaptor and test have larger aftereffects than those with dissimilar (comparing the left column of graphs with the right).

**Figure 8:**
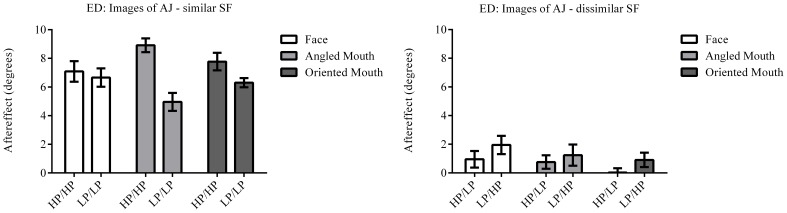
**Aftereffect magnitudes for Observer ED for stimuli derived from a different face: AJ**. These data are from a repetition of the experiment using stimuli derived from a different face with the same transformations applied. The magnitudes of aftereffects are similar to those shown in Figure 7, demonstrating the patterns of results are not specific to a single face.

The column of graphs on the left (right) of Figures 3–5 and 6 shows data for the LP (HP) test pattern conditions as previously described. The top pair of graphs in the figures shows the results for the face stimuli for the observers. The ordinate represents the probability of responding that a face is happy and the abscissa the displacement in angle of the mouth of the test pattern (positive indicates deformation toward smiling and negative toward frowning). The legend indicates the adaptation condition. Data pertaining to LP and HP adaptors are plotted in blue and red, respectively. Adapting conditions that are labeled +15 are happy and −15 sad. The functions fitted to the data of the happy (sad) adaptor conditions are solid (dashed) lines. It is immediately evident that after adaptation to a happy (sad) face a neutral face is reported to appear sad (happy) in more than half of the trials. In order for a test face to be reported happy (sad) in an equal proportion of trials, the PSE, it must be transformed toward happy (sad). The aftereffect causes the test face to be perceived as more different to the adaptor face than it actually is. The same effect is observed for adaptor and test face pairs that are both HP, and both LP in spatial frequency. The middle and bottom pairs of graphs show the comparable results for the angled and oriented mouth stimuli, respectively. For the angled mouth stimuli positive (negative) deformations in angle produces stimuli that are concave to the left (right). Observers were required to report whether the test stimuli were concave to the left or right. It is clear from the data that adaptation to a stimulus that is concave to the left, for example, results in a decrease in the probability of reporting that the test stimuli are concave to the left. For the oriented mouth stimuli a positive deformation in angle results in the stimulus being oriented anti-clockwise of vertical. Observers were required to report whether the test stimuli were oriented anti-clockwise or clockwise of vertical. Adaptation to a stimulus with a positive deformation in angle resulted in a reduction in the probability of reporting that the test stimuli were oriented anti-clockwise of vertical. The common qualitative result across the three stimulus types is that adaptation results in repulsive aftereffects. In order to compare the aftereffects quantitatively the magnitudes of the effects, measured as the differences between the points of subjective equality for adaptors with different signs of deformation in angle (+15 and −15) were calculated, and are presented in Figure 7. The column of graphs on the left (right) show the magnitudes of the aftereffect for test stimulus pairs that were of similar, that is HP/HP or LP/LP (dissimilar, HP/LP or LP/HP) spatial frequency. The bottom row of graphs shows the averaged data of the four observers. Figure 8 shows the results for a repeat of the experiment using a different face, AJ, transformed in the same manner as the image of VB.

The data summarized in the bottom row of graphs of Figure 7 were tested statistically. A two-tailed, paired *t*-test of the data of the four observers showed that the aftereffect magnitudes for the conditions with similar spatial frequencies in the adaptor and test (the left hand column of graphs) did not differ across conditions where the adaptor and test patterns were both high (HP/HP) frequency, or both low (LP/LP) frequency (*p* = 0.9294, *t*_(11)_ = 0.09066). The means of these populations were 5.568 ± 0.939 (95% CI) and 5.532 ± 0.549 degrees, respectively. These conditions were, therefore, combined within stimulus types into conditions of similar spatial frequency in test and adaptor for further analysis. Furthermore, a paired *t*-test of the aftereffect sizes for conditions with dissimilar spatial frequencies across adaptor and test (HP/LP or LP/HP; the right hand column of graphs) did not differ in the order of the frequencies used (*p* = 0.9689, *t*_(11)_ = 0.03994). The means for these populations were 2.430 ± 0.488 and 2.415 ± 0.824 degrees, respectively. These conditions were also combined within stimulus types into conditions of dissimilar spatial frequency. The aforementioned means show that the aftereffect magnitudes for conditions where the adaptor and test had spatial frequencies in the same range were greater than twice those where their spatial frequencies were in different ranges. The magnitudes for the conditions with dissimilar spatial frequencies were, however, also significantly greater than zero (a result that might be attributed to second-order tilt aftereffects or a bandwidth for the spatial frequency channels that was broader than the band separating the HP and LP spatial frequency ranges). Following these amalgamations of conditions we are left with six conditions to compare, these being face, angled mouth and oriented mouth stimulus types with similar or dissimilar spatial frequencies in the adaptor and test patterns. A One-Way ANOVA incorporating Tukey's multiple comparisons test was used to compare the magnitudes of the aftereffects within these populations. The results of the multiple comparisons test are reported in Table 1.

**Table 1:**
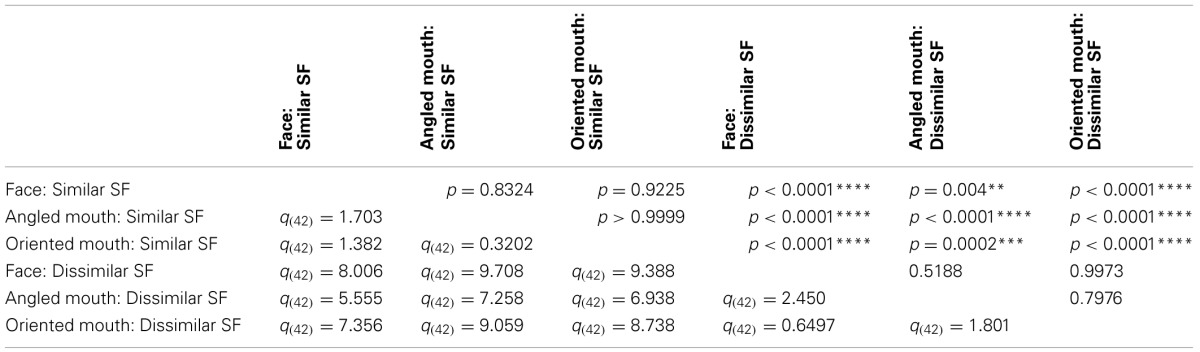
**This table compares the magnitudes of the aftereffects across six conditions**.

The conditions are face, angled mouth and linear orientated mouth stimuli with similar and dissimilar spatial frequency (SF) content in the adaptor and test pairs. For example the condition Face: Dissimilar SF represents data pertaining to adaptor-test face stimulus pairs with dissimilar spatial frequency content. That is, a high-pass (HP) adaptor face and low-pass (LP) test face (HP/LP) or a LP adaptor face and HP test face (LP/HP). The data for each condition are compared with the data for each of the other five conditions using an ANOVA with a tukey multiple comparisons test. The q-values for the tukey test are entered below the major diagonal and the corresponding p-values above. Four asterisks denote p < 0.0001, three p < 0.001, and two p < 0.01. The top right hand quadrant of data cells all show significant differences between the aftereffect magnitudes for these pairings of conditions. These pairings represent all of the comparisons between a condition with similar SF content in the adaptor and test and a condition with dissimilar SF content. All other comparisons (similar vs similar and dissimilar vs dissimilar SF content) are not significantly different whatever the pairing of stimulus type (face with oriented mouth for example).

To summarize the results reported in Table 1, the magnitudes of the aftereffects were only significantly different when pairwise comparisons were made between a condition with similar spatial frequency content across adaptor and test stimulus and a condition with dissimilar spatial frequency content across adaptor and test. All three stimulus types; faces, angled mouths and oriented mouths, show the same dependency on the similarity between the spatial frequency content of adaptor and test.

## Discussion

The results of this study demonstrate that adaptation to a happy face causes a face with a neutral expression to look sad and vice versa. This result has been demonstrated in the past (Xu et al., 2008). The expression of the face is in this instance, however, entirely dictated by the shape of the mouth and the magnitude and direction of the aftereffect can be predicted by an angle aftereffect introduced into a straight mouth, abstracted from the face, by adaptation to the manipulated angled mouth used in the face adaptor. In turn, the angle aftereffect can be predicted by the tilt aftereffect introduced into a vertically oriented mouth by an oriented mouth tilted from the vertical. Moreover, the spatial frequency specificity of these three effects is the same, pointing to the same, low level, adaptation effect being responsible for all three. We propose that the results can all be understood as due to a reduction in sensitivity in the orientation selective neurons of the primary visual cortex that were stimulated during adaptation. When adaptor and test orientations are different, the response of the population of neurons sensitive to the whole range of orientations is biased toward neurons that were not previously stimulated, that is those whose preferred orientations are more different from the adaptor than the test. The resultant vector sum of the activity in the population of neurons with the complete range of preferred orientation is therefore skewed giving rise to the tilt aftereffect (Bekesy, 1929; Gilbert and Wiesel, 1990; Clifford et al., 2001; Clifford, 2002; Dickinson et al., 2012a,b). The tilt aftereffect is a local and retinotopic phenomenon (Knapen et al., 2010) but it has been shown that its systematic application over space in a tilt aftereffect field can provide an explanation for complex shape aftereffects including, perhaps, face shape aftereffects (Dickinson et al., 2010). It is recognized that the aftereffects apparent at one level of visual coding might be inherited from adaptation at lower levels, but Dickinson et al. (2010) was the first explicit demonstration that complex shape aftereffects could be wholly accounted for by a spatially extended field representing the tilt aftereffect experienced locally. Dickinson et al. (2012a) showed that the same shape aftereffects could be predicted by a local population encoding of orientation within a tilt aftereffect field. Dickinson et al. (2010) proposed that a tilt aftereffect field could account for face aftereffects. The current study is totally consistent with this interpretation, showing that a change in morphology of the face due to a local tilt aftereffect can account for the change necessary to allow reliable reporting of the demeanor of a face. This conjectured explanation for face aftereffects makes strong testable predictions. It also predicts some of the controversies currently unresolved in face processing literature. For example, currently under discussion is whether faces are identified by explicit neural templates or by reference to a norm. This dichotomy is prompted by the differing representations of orientation (or spatial scale) and color at a local level (Webster, 2012). Orientation, as we have discussed, is represented by a continuum of orientation channels, while saturation of a color increases monotonically from a neutral gray. The argument is made that similar principles might underlie representations at successive levels of visual processing and, therefore, that we might expect the effects of adaptation to reveal the representation used for a particular visual stimulus. Adaptation within a channel based system might be expected to produce aftereffects that produce repulsion of perceptual representation away from the adaptor whilst adaptation within a norm based system might produce a displacement of the norm. Dickinson et al. (2010), using an adapting face distorted by a sinusoidal modulation of radius, demonstrated that the magnitude of the face distortion aftereffect increases with the amplitude of distortion of the adaptor to a point, and then decreased beyond this amplitude. This might be interpreted as an indication that face morphology is encoded in a channel based fashion. The position of the rollover of the size of the aftereffect with respect to the maximum orientation difference introduced by the distortion of the face, however, was consistent with the maximum in the curve that describes the tilt aftereffect as a function of orientation difference. We suggest, therefore, that face aftereffects that depend upon differences in the morphology of adapting and test faces can often be predicted by a tilt aftereffect field. Certain manipulations of face stimuli have been developed, however, that might not yield to a tilt aftereffect field explanation, for example those manipulating eye height (Susilo et al., 2010). We suggest that other local field explanations such as local spatial scale or aspect ratio adaptation might account for these.

A tilt aftereffect field explanation for shape adaptation, of course, is agnostic to the representation of the higher level properties of the visual stimulus. This is not to say that adaptation does not occur in higher level but that morphological aftereffects are unlikely to be of any value in elucidating the mechanisms of representation of high level stimulus properties, unless aftereffects that are demonstrably not local can be identified. The inheritance of the effects of adaptation in lower levels, though, does offer a potential solution to the vexed question of what purpose the aftereffects serve. Because the mechanisms pertaining to the higher levels of shape analysis are invariant under the effects of adaptation at lower levels, the tilt aftereffect field provides a general mechanism for exaggerating the perceived difference in the higher level stimulus properties of successively presented stimuli. The state of adaptation is shown in this study to be rapidly acquired and large thereby rendering successively experienced facial expressions more perceptually different than they otherwise would be.

Having proposed this model it has to be conceded that this view and methodology is unconventional. Rather than attempting to demonstrate that lower-level aftereffects can account for the change in a higher-level percept following adaptation, conventional studies typically use presumed properties of low-level effects to devise experiments that mitigate these effects. It has been argued that some aftereffects, rather than being retinotopic, are spatiotopic or even position invariant. If the tilt aftereffect is considered to act locally in a retinotopic manner then any systematic aftereffects that were not retinotopic could be assumed to be high-level. Evidence, however, is equivocal. Melcher (2005), for example, claimed that face, form and tilt aftereffects were spatiotopic, while Knapen et al. (2010) reported that the tilt aftereffect was constrained to retinotopic coordinates. These results are, obviously, mutually exclusive. Dickinson et al. (2012b), however, showed that rapidly acquired shape and face aftereffects are retinotopic which may suggest a resolution to this conflict. If the effects of adaptation are accumulated at a point on the retina, as suggested by the results of Blakemore and Over (1974), then the aftereffect experienced at a point in space would be dependent on the history of retinotopic adaptation over eye movements. The experimental paradigm employed by Dickinson et al. (2012b), using an adaptation time of 160 ms, precluded eye movements during adaptation and, therefore, the retinotopic aftereffects revealed might be assumed to indicate that the presumed high-level aftereffects of other experiments, that purported to control for low-level aftereffects, could in fact arise from spatially distributed retinotopic low-level aftereffects accumulated over successive eye movements during adaptation. Other attempts to mitigate low-level effects, for example by introducing a mismatch in size of adaptor and test might be compromised by the same effect. Even in the absence of these accumulation effects the assumption that a spatial mismatch of adaptor and test entirely mitigates low-level effects is erroneous. Local differences in orientation would exist and those differences would be expected to produce tilt aftereffects. The effects would be different to those experienced for spatially matched conditions, and for certain transformations of adaptor and test might not be expected to systematically bias the judgment made (see Dickinson et al., 2010), but they would exist nonetheless. It is often assumed that the local aftereffects are totally eliminated by controls for low-level effects, and that the residual adaptation is, therefore, due to high-level adaptation, but perhaps it is more likely that the controls only reduce the low-level effects.

A novel recent paper exploited the phenomenon of crowding in an effort to dissociate aftereffects of orientation and facial expression (Xu et al., 2012). The stimuli consisted of curved lines, or cartoon faces incorporating the same curved line as a mouth. The curved line either made the cartoon face smile or frown. Adaptation effects were studied both within and across these stimulus types (with the curves retinotopically coincident). It was found that the crowding effect of curves flanking an adapting curve reduced the curvature aftereffect more than the facial expression aftereffect. Conversely, crowding of the adapting face with flanking faces reduced the facial expression aftereffect more than the curvature aftereffect. These effects are indeed consistent with the predicted specificity of crowding at the higher and lower levels of representation of the stimuli, but it is still possible to speculate that the different conditions of crowding might have a differential effect on involuntary eye movements. In conclusion, although there is some evidence to suggest that controls for low-level aftereffects leave some residual aftereffect that might be attributed to high-level adaptation the results of this study demonstrate that under certain circumstances high-level aftereffects can be wholly accounted for by inheritance of low-level aftereffects. We, therefore, advise caution in the presumption of knowledge of the locus of the psychologist's microelectrode when performing adaptation studies.

### Conflict of interest statement

The authors declare that the research was conducted in the absence of any commercial or financial relationships that could be construed as a potential conflict of interest.
